# Real‐Time, Inline Quantitative MRI Enabled by Scanner‐Integrated Machine Learning: A Proof of Principle With NODDI

**DOI:** 10.1002/mrm.70388

**Published:** 2026-05-05

**Authors:** Samuel Rot, Iulius Dragonu, Christina Triantafyllou, Matthew Grech‐Sollars, Anastasia Papadaki, Laura Mancini, Stephen Wastling, Jennifer Steeden, John S. Thornton, Tarek Yousry, Claudia A. M. Gandini Wheeler‐Kingshott, David L. Thomas, Daniel C. Alexander, Hui Zhang

**Affiliations:** ^1^ Hawkes Institute and Department of Computer Science UCL London UK; ^2^ NMR Research Unit, Queen Square MS Centre, Department of Neuroinflammation, UCL Queen Square Institute of Neurology, Faculty of Brain Sciences UCL London UK; ^3^ Research and Collaborations GBI Siemens Healthcare Ltd Camberley UK; ^4^ Lysholm Department of Neuroradiology, National Hospital for Neurology and Neurosurgery University College London Hospitals NHS Foundation Trust London UK; ^5^ Neuroradiological Academic Unit, Dept of Translational Neuroscience and Stroke, UCL Queen Square Institute of Neurology UCL London UK; ^6^ Centre for Cardiovascular Imaging, Institute of Cardiovascular Science UCL London UK; ^7^ Department of Brain & Behavioural Sciences University of Pavia Pavia Italy; ^8^ Digital Neuroscience Centre IRCCS Mondino Foundation Pavia Italy

**Keywords:** diffusion MRI, inline reconstruction, machine learning, neural networks, NODDI, quantitative MRI

## Abstract

**Purpose:**

The clinical feasibility and translation of many advanced quantitative MRI (qMRI) techniques are inhibited by their restriction to ‘research mode’, due to resource‐intensive, offline parameter estimation. This work aimed to achieve ‘clinical mode’ qMRI, by real‐time, inline parameter estimation with a trained neural network (NN) fully integrated into a vendor's image reconstruction environment, therefore facilitating and encouraging clinical adoption of advanced qMRI techniques.

**Methods:**

The Siemens Image Calculation Environment (ICE) pipeline was customized to deploy trained NNs for advanced diffusion MRI parameter estimation with Open Neural Network Exchange (ONNX) Runtime. Two fully‐connected NNs were trained offline with data synthesized with the neurite orientation dispersion and density imaging (NODDI) model, using either conventionally estimated (NN_MLE_) or ground truth (NN_GT_) parameters as training labels. The strategy was demonstrated online in two healthy volunteers (one rescanned) and evaluated offline with synthetic data, testing two diffusion protocols.

**Results:**

NNs were successfully integrated and deployed natively in ICE, performing inline, whole‐brain, in vivo NODDI parameter estimation in < 10 s. The proposed workflow was reproducible across protocols, volunteers and rescans. DICOM parametric maps were exported from the scanner for further analyses. Comparisons between NN_MLE_ and NN_GT_ suggested NN_MLE_ parameter estimates to be more consistent with conventional fitting, a finding supported by offline evaluations.

**Conclusion:**

Real‐time, inline parameter estimation with the proposed generalizable framework resolves a key practical barrier to the potential clinical uptake of advanced qMRI methods, enabling their efficient integration into clinical workflows. Next steps include incorporation of pre‐processing methods and evaluation in pathology.

## 1 Introduction

Quantitative MRI (qMRI) enables the estimation of tissue properties of interest using biophysical models that relate these properties to measured MR signals. Compared to conventional MRI, it provides biomarkers that have less dependence on acquisition settings and may better inform on pathology. One such advanced qMRI technique is Neurite Orientation Dispersion and Density Imaging (NODDI), a three‐compartment model of multi‐shell diffusion MRI signals [1]. As a brain microstructure imaging method, NODDI provides voxel‐wise estimates of the orientation dispersion index (ODI, degree of spreading of neurites), the neurite density index (NDI, the intra‐neurite fraction of tissue), and the free water fraction (FWF, the volume fraction of bulk liquid undergoing free diffusion), as well as the predominant neurite orientations.

Although promising in research settings [2], qMRI methods have not yet reached “clinical maturity”, according to Granziera et al. [3], with multi‐shell diffusion models such as NODDI further from adoption than other techniques. One key reason, they believe, is that the methods and tools to reconstruct parametric maps are not clinically available [3]. Indeed, parameter estimation is typically done offline. Often this requires exporting image data from MRI systems onto high‐performance research workstations where, depending on model complexity, conventional parameter estimation may take multiple hours [1], though strategies for acceleration exist [4]. Although it has been argued that a lack of accumulated evidence and validation, rather than computational costs, is the primary inhibitor to clinical adoption of techniques like NODDI [2], we believe that the computational costs themselves are a fundamental reason for this lack, restricting large‐scale clinical research and validation studies. They also still render many potential applications in acute or point‐of‐care scenarios categorically infeasible. Even when lengthy reconstruction times are acceptable, the need for external computing resources is disruptive, and parametric maps seldom re‐enter the clinical data stream, failing to reach reporting systems. Recently, 50% of surveyed European neuroradiologists suggested that technical improvements in software and hardware would catalyze greater uptake of qMRI [5]; a lack of processing software (23%) and time intensive processes (39%) were identified as major impediments [5]. Altogether, we believe that the entire conventional qMRI workflow is impractical and incompatible with clinical practice, holding advanced techniques back in ‘research mode.’ Fortunately, two exciting recent advances with machine learning (ML) promise to make qMRI more clinically viable.

First, ML is revolutionizing parameter estimation: in particular, fully‐connected neural networks (NN) trained with synthetic data can replace computationally expensive model fitting procedures, producing voxel‐wise parameter estimates near instantaneously [6, 7, 8, 9, 10, 11]. An additional advantage is the potential to resolve model (parameter) degeneracy [12, 13], which occurs when multiple parameter combinations produce indistinguishable noisy signals. ML approaches are typically either supervised [6, 9] or self‐supervised [10] (unsupervised [8]). The former utilizes ground truth (GT) parameters as targets (labels) during training, computing and minimizing the loss in parameter space; the latter self‐generates labels to minimize the loss in signal space. Supervised learning is a flexible and intuitive approach but may lead to biased parameter estimates, whereas self‐supervised learning may yield more accurate parameter estimates, at the cost of implementational challenges [7]. To overcome this trade‐off, Epstein et al. recently proposed supervised learning with non‐GT target parameters, instead utilizing the estimates returned by a conventional fit of the synthetic data [7]. This strategy was shown to emulate the improved accuracy of self‐supervised learning, while retaining the scalability and simplicity of a supervised framework.

Second, MRI vendors have recently begun to support inline inference with trained NNs for image manipulation tasks. For example, the Siemens Framework for Image Reconstruction Environments (FIRE) prototype [14] permits a containerized deployment of trained NNs [15, 16], interfacing with the reconstruction pipeline using the open‐source ISMRM raw data format [17]. Complete integration of trained NNs into the vendors' native image reconstruction pipeline is also possible, using C or C++ runtime libraries [18, 19, 20, 21]. Otherwise, external third‐party reconstruction frameworks may be configured for inline deployment of trained NNs (e.g., Gadgetron [22] with the InlineAI module [23, 24]), however this still demands dedicated computing and research infrastructures, which clinical settings may be unable to accommodate.

The primary aim of this work was to exploit and combine the two outlined advances to achieve real‐time, inline, fully scanner‐integrated qMRI parameter estimation with ML. We chose to demonstrate this strategy for NODDI, with the secondary aim of applying and validating Epstein et al.'s novel supervised ML qMRI model fitting approach [7] for reduced parameter estimation bias without the complexity of self‐supervised learning.

## 2 Methods

### 2.1 Workflow Overview

**FIGURE 1:**
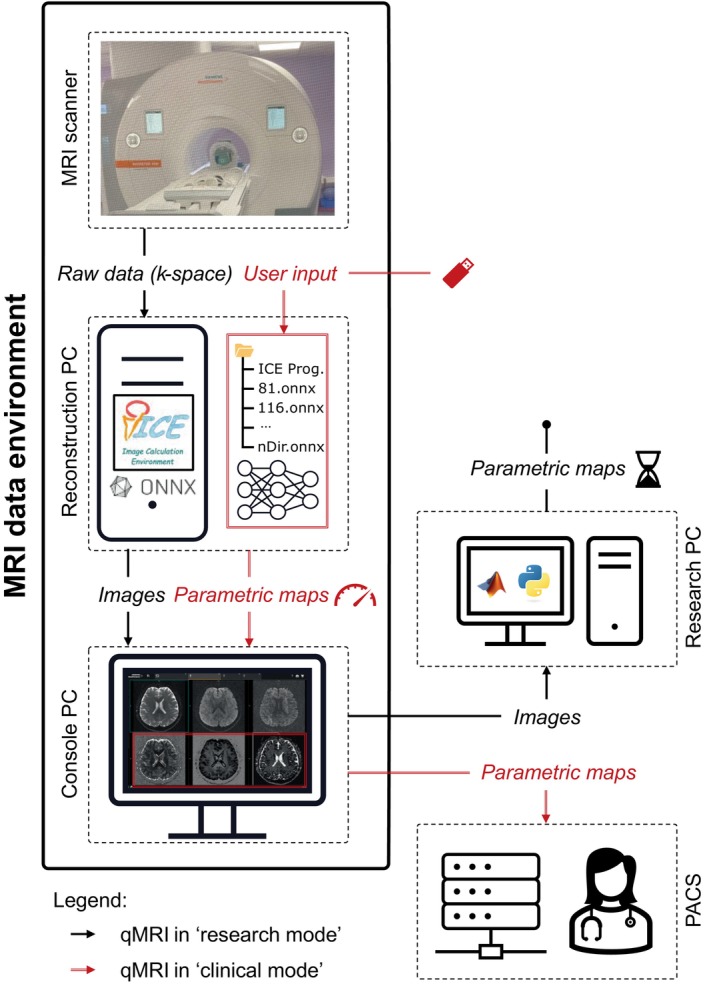
A summary schematic of the proposed workflow for qMRI in ‘clinical mode’, contrasted against a conventional workflow for qMRI in ‘research mode’. Raw k‐space data are sent from the scanner to the vendor's reconstruction server and reconstructed into images slice by slice. From this point, the two workflows differ. In ‘research mode’ qMRI: images are displayed on the scanner console PC and exported for offline qMRI parameter estimation, usually onto a high‐performance research workstation, to meet the high computational demands of parameter fitting. Once estimated, parametric maps often remain within the research environment, as a transfer back onto the scanner console PC is cumbersome. In the proposed ‘clinical mode’ qMRI, still on the reconstruction server, slices and multiple image data dimensions are accumulated and fed through an integrated trained neural network (NN) for real‐time inference of qMRI model parameters. The resulting parametric maps are stored in DICOM format and sent to the console PC for display. From there, they may be forwarded to PACS, or other clinical reporting systems, alongside conventional diagnostic imaging data. A one‐time user import of ICE program files and ONNX trained NNs is required, for example, via flash drive. A simple NN naming scheme, like using the number of diffusion encodings, allows automatic selection and concurrent storage of different NNs.

To introduce the core of our method, Figure 1 illustrates the proposed ‘clinical mode’ workflow for qMRI, highlighting key differences from the conventional ‘research mode’ approach, including real‐time neural network (NN) based parameter estimation using the scanner's computing hardware, and integration with clinical reporting systems.

Inline NN inference was performed using ONNX (Open Neural Network Exchange) Runtime [25] libraries (https://www.nuget.org/packages/Microsoft.ML.OnnxRuntime), which were compiled into a custom reconstruction program developed in Siemens' Image Calculation Environment (ICE), using C++. The ICE program first accumulates images into a 4D data structure as they pass through the reconstructor as usual, slice‐by‐slice. Once all images have been reconstructed and the accumulated data structure is complete, volumes are normalized by the mean *b* = 0 s/mm^2^ signal in each voxel. Inference is then performed voxel‐wise, on the reconstruction server's CPU, with a trained NN in the ONNX format, as supplied by the user. The resultant parametric maps of ODI, NDI and FWF are finally output as a separate DICOM series, with floating point numbers stored as 12‐bit integers scaled by 1000, achieving a precision of three decimal places. Parametric maps may be visualized, analyzed and inspected natively on the console PC and sent directly to reporting systems for clinical evaluation.

The following subsections detail how NNs were trained, deployed in vivo, and evaluated.

### 2.2 Neural Network Development

**FIGURE 2:**
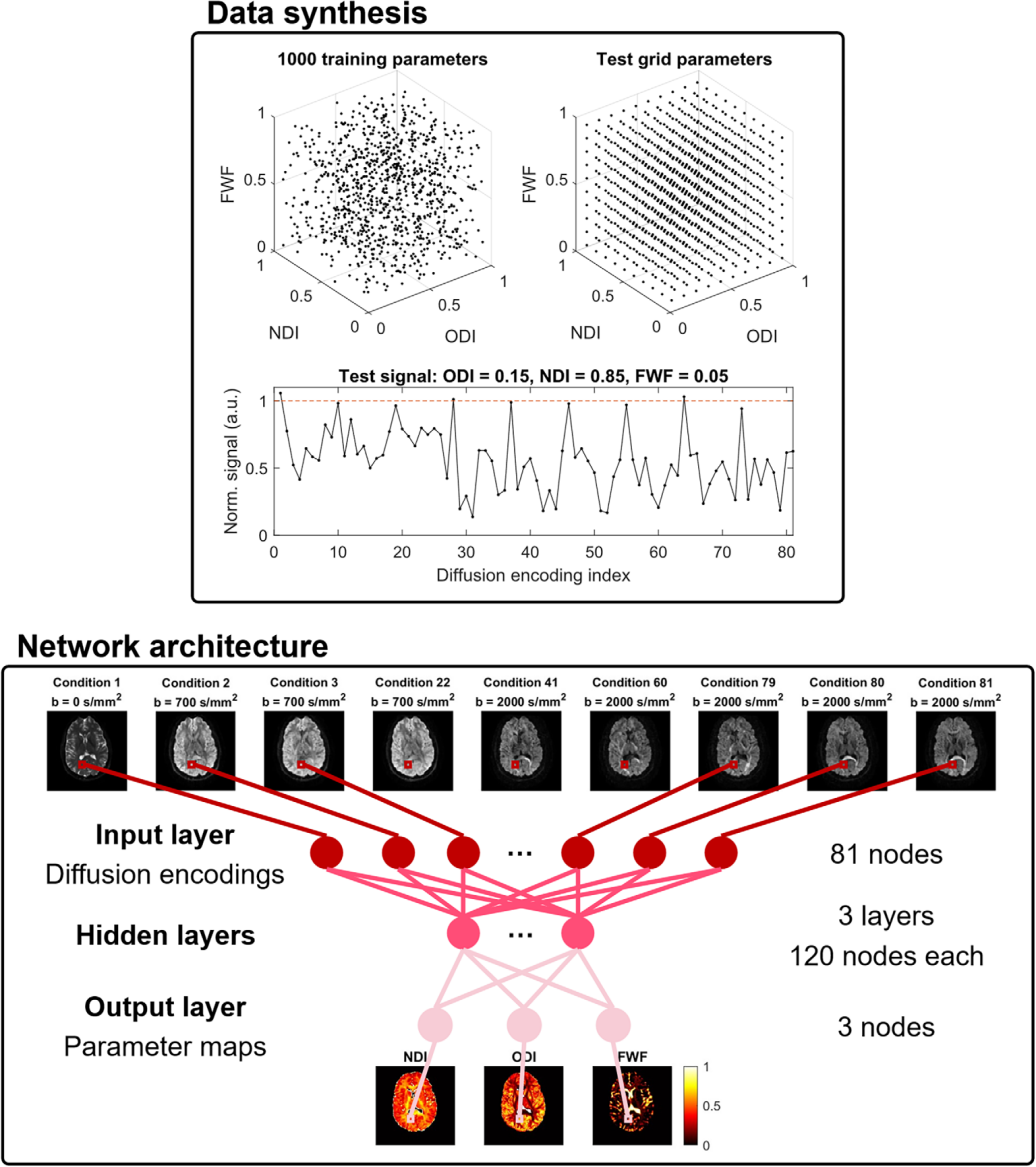
The upper panel shows training and test data parameters in 3D parameter space of orientation dispersion index (ODI), neurite density index (NDI), free water fraction (FWF). For each grid point of test parameters, 100 signals with unique noise were synthesized, with an example shown for typical white matter parameters (high NDI, low ODI, and low FWF). The lower panel shows a schematic of the fully connected neural network (NN) architecture, employed in a voxel‐wise manner, and demonstrated for the two‐shell protocol (the number of input nodes is protocol dependent). Images in the schematic are from example data of the NODDI MATLAB Toolbox.

Neural networks (NNs) were developed and trained offline. Operating on a voxel‐wise basis, NNs receive as input a diffusion‐encoded signal vector and output a corresponding parameter vector (summarized in Figure 2). This setup enables training using synthetic diffusion MRI signals. First, ground truth (GT) parameter values for orientation dispersion index (ODI), neurite density index (NDI), free water fraction (FWF) were drawn from random uniform distributions [6], U (Figure 2) for 2^18^ unique combinations (or training ‘datasets’):

ODI∼U(0.05,1.0);NDI∼U(0.05,1.0);FWF∼U(0.0,0.95)

Distribution limits were chosen to reduce parameter degeneracies. Each parameter combination was allocated a fiber orientation, drawn from a random uniform distribution over the unit sphere [26]. With each unique combination of GT parameters, a diffusion encoded signal was synthesized with the forward NODDI model using an internal version of the NODDI MATLAB Toolbox (NMT, http://mig.cs.ucl.ac.uk/index.php?n=Tutorial.NODDImatlab). To demonstrate adaptability to multiple protocols, signals were synthesized for two‐shell (81 encodings) and three‐shell (116 encodings) diffusion schemes (Table S1). Signals were normalized to *b* = 0 s/mm^2^ and complex Gaussian noise of SNR = 15 at *b* = 0 s/mm^2^ (representative of white matter [WM] in vivo) was added before calculating the magnitude, resulting in Rician‐distributed measurements.

Training data were fitted conventionally with a maximum likelihood estimator (MLE) using the NMT. To accelerate fitting, the grid search across parameter space was skipped, directly supplying GTs to initialize the gradient descent algorithm. Following Epstein et al. [7], MLE parameters were then utilized as training labels. For comparison, a separate supervised NN was also trained with GT training labels. The two NNs are herein referred to as NN_MLE_ and NN_GT_. They were implemented in PyTorch [27] (version 2.2.1) with the fully connected architecture illustrated in Figure 2 and summarized in Table S2, together with training parameters. The output layer utilized a hard sigmoid activation function to constrain the NN output between 0 and 1. After training, the best model, with lowest validation loss, was saved for evaluation.

### 2.3 In Vivo Demonstration

Full online operation was tested on a MAGNETOM Vida 3T system (Siemens Healthineers, Forchheim, Germany). With informed consent and approval of the local ethics committee, two healthy volunteers (V1: male, 31 years; V2: male, 25 years) were imaged using both diffusion protocols in Table S1. V2 was rescanned without repositioning, yielding a total of six diffusion imaging datasets. After transferring the ONNX files of trained NNs onto the console PC, the custom ICE program was used to reconstruct the raw data with trained NNs inline. Parametric maps and diffusion encoded images were exported in DICOM format. After offline conversion to NIFTI format, parametric maps were also reconstructed on a research workstation with a conventional MLE routine using the NMT, without any additional pre‐processing. For tissue specific quantitative analyses, masks for each image series were obtained utilizing SynthSeg [28] on mean *b* = 0 s/mm^2^ images and thresholding the probabilistic mask at 0.95 for WM, gray matter (GM) and CSF (ventricles).

### 2.4 Synthetic NN Evaluation

To address the secondary aim of this work, trained NNs were evaluated with synthetic test data. 3D parameter space was sampled uniformly within 0.05–0.95 for ODI, NDI and FWF. GT parameters were spaced at increments of 0.1, giving P=1000 unique parameter combinations, as shown in Figure 2. Then, to investigate estimation accuracy and variability, test signals were synthesized with N=100 unique noise realizations for each parameter combination, for a total of 100 000 test ‘datasets’. To control for variations due to the fiber orientation, it was fixed to [x,y,z]=[1,0,0].

To prevent degenerate parameter estimates (e.g., ODI and NDI for high FWF) from skewing or biasing quantitative analyses, a degeneracy‐free subspace, representative of tissue (WM and GM), was defined: 0.05–0.55 (ODI), 0.35–0.85 (NDI), 0.05–0.45 (FWF). Model performance was evaluated at each GT parameter grid point, $x_{p}=\left[\mathrm{ODI},\mathrm{NDI},\mathrm{FWF}\right]$, by computing the mean, $\mu_{p}$, bias, $b_{p}$, and standard deviation, $\sigma_{p}$, of fitted parameters across repeats:

$$\mu_{p}=\frac{1}{N}\sum_{n=1}^{N}{\hat{x}}_{\mathrm{pn}};b_{p}=\mu_{p}-x_{p};\sigma_{p}=\sqrt{\frac{\sum_{n=1}^{N}{\left({\hat{x}}_{\mathrm{pn}}-\mu_{p}\right)}^{2}}{N}}$$

where ${\hat{x}}_{\mathrm{np}}$ are the estimated parameters of noise realization n and grid point p. As summary metrics, the mean of magnitude biases, |b|, and the mean of the absolute differences ($\bar{\left|\Delta \right|}$) of paired NN‐MLE estimates from a conventional fit, were calculated as follows:

$$\bar{\left|b\right|}=\frac{1}{P}\sum_{p=1}^{P}\left|b_{p}\right|\text{;}\bar{\left|\Delta \right|}=\frac{1}{\mathrm{PN}}\sum_{p=1}^{P}\sum_{n=1}^{N}\left|{\hat{x}}_{pn}^{\mathrm{NN}}-{\hat{x}}_{pn}^{\mathrm{MLE}}\right|$$

## 3 Results

**FIGURE 3:**
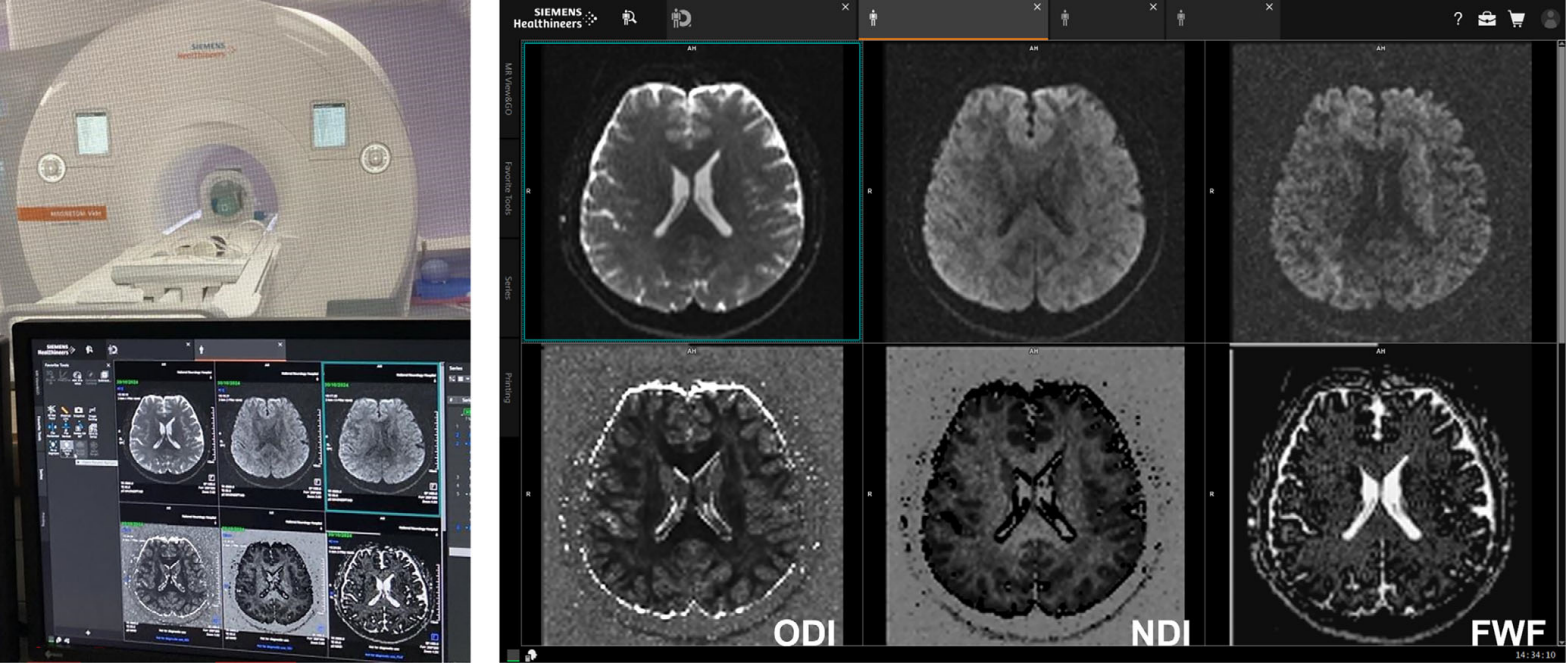
Right, a screenshot of the MAGNETOM Vida 3T (Siemens Healthineers, Forchheim, Germany) console monitor, showing inline reconstructed maps (ODI, NDI, FWF from left to right, bottom row) for a participant scanned. Sample diffusion encoded images are shown in the top row. Left shows a photograph of the console from the control room.

In vivo NODDI parametric maps were estimated inline and displayed on the scanner console PC, demonstrating successful integration of the NN into the scanner's reconstruction environment (Figure 3). The inline reconstruction for the entire 4D dataset took under 10 s.

**FIGURE 4:**
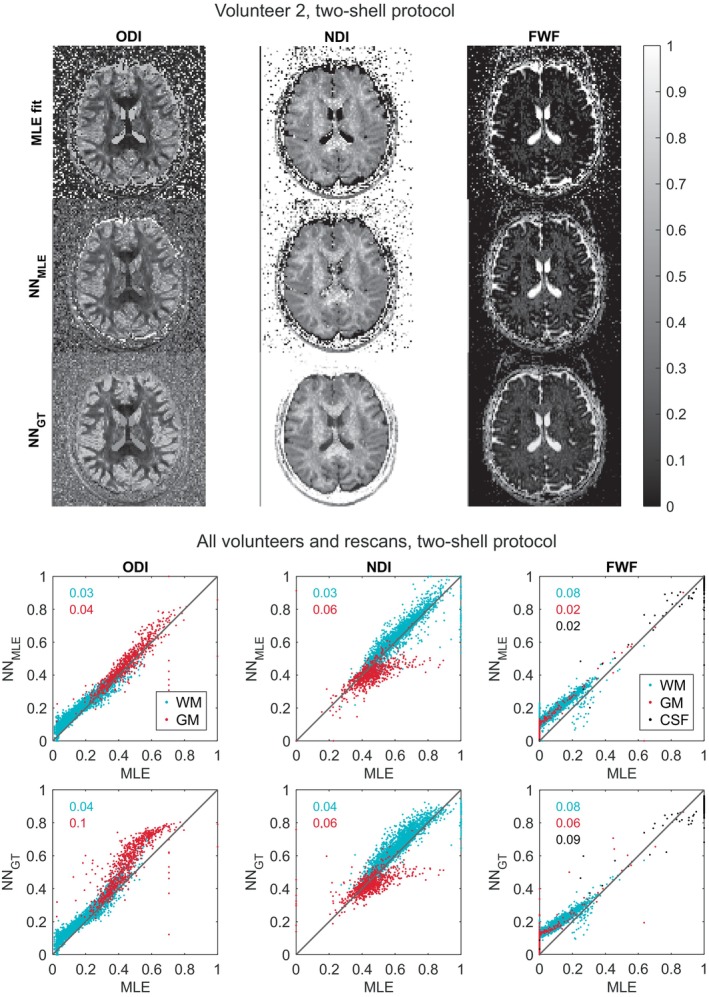
Estimated orientation dispersion index (ODI), neurite density index (NDI) and free water fraction (FWF) parameter maps for an axial slice of V2 and the two‐shell protocol. The upper row shows maps fitted conventionally with the NODDI MATLAB Toolbox (indicated MLE); the next two rows show maps inferred with the two trained neural networks (NN_MLE_ and NN_GT_). The bottom two rows show scatter plots of estimated parameters for each tissue type of single axial slices of all volunteers and rescans using the two‐shell protocol, comparing MLE to NN_MLE_ (row 4) and NN_GT_ (row 5). Pairwise MLE‐NN mean absolute differences for each tissue class are noted in the top left of each panel. NN parameter maps were exported as DICOMs, converted to NIFTI format and rescaled from integer (range 0–1000) to float (range 0–1). No post‐processing was performed.

In vivo parametric maps were exported in DICOM format for analysis. They are displayed in Figure 4 for V2 and the two‐shell protocol (see Figure S1 for the three‐shell protocol) and compared to parametric maps obtained with a conventional MLE fit of an axial slice. On a research workstation equipped with an Intel Xeon w3‐2435 CPU, the MLE fit took approximately 3 min for an individual masked slice, using 6 parallel processes. This scales to approximately 2 h for a whole, masked brain (˜0.04 s per voxel). Parametric maps for all volunteers, rescans and protocols, as estimated with NN_MLE_, are shown in Figure S2.

Also displayed in Figure 4 are scatter plots of estimated parameters in different tissue types (combining all volunteers and rescans, two‐shell protocol), comparing conventional parameter estimation to NN_MLE_ and NN_GT_. A complete reference of tissue‐wise parameter estimates for all volunteers and rescans, protocols and estimation methods is provided in Table S3. NN_MLE_ returns slightly less biased estimates, more consistent with MLE, in WM (NDI, FWF), GM (ODI, FWF), and CSF (FWF), as substantiated by smaller mean absolute differences between paired NN‐MLE estimates for NN_MLE_. Moreover, Figure 4 is reproduced for the three‐shell protocol in Figure S1, with similar but slightly improved patterns of bias and agreement of NN_MLE_ and NN_GT_ (in WM).

**FIGURE 5:**
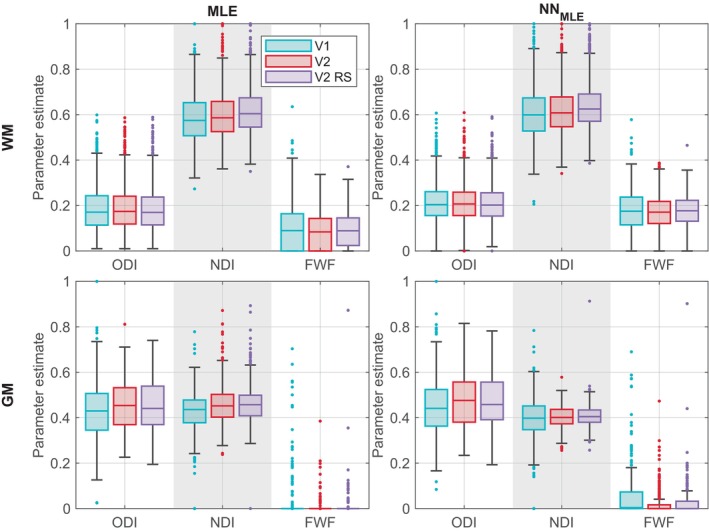
Boxplots of estimated orientation dispersion index (ODI), neurite density index (NDI), and free water fraction (FWF) parameters for an axial slice across all volunteers and rescans (different colors) of the two‐shell protocol. Each panel shows data for a particular tissue type and estimation method, as indicated in row and column labels. The solid horizontal line indicates the median, the box indicates the interquartile range (IQR) and whiskers indicate outlier bounds, 1.5 IQRs from the lower and upper quartiles.

Figure 5 summarizes the distributions of tissue‐wise parameter estimates across volunteers and rescans. Estimates appear in general agreement, and small deviations are consistent across methods (e.g., slightly increased NDI in WM for V2 rescan). ODI, FWF and WM appear more stable than NDI and GM. Figure 5 is reproduced for the three‐shell protocol in Figure S3.

The offline evaluation of trained NNs with synthetic test data is summarized in Figure S4, showing vector plots of the bias and line plots of the standard deviation of estimated test parameters, as well as scatter plots comparing MLE to NN_MLE_ and NN_GT_ estimates, all within a tissue parameter subspace. NN_MLE_ and MLE bias vectors closely overlap, indicating comparable estimation biases, whereas NN_GT_ bias vectors extend and deviate more from the GT. Mean magnitude biases ($\bar{\left|b\right|}$) were 0.029, 0.034, and 0.075 (Figure S4) for MLE, NN_MLE_ and NN_GT_ respectively. In scatter plots, NN_GT_ also returns a greater point spread, away from the line of identity, as reflected quantitatively by increased mean absolute differences. The same visualizations are reproduced for the three‐shell protocol in Figure S5. Additional diffusion encodings reduce overall bias, as well as the difference between NN_MLE_ and NN_GT_.

## 4 Discussion

This study successfully demonstrated the possibility of scanner‐integrated, real‐time qMRI by leveraging ML methods, resolving a key practical barrier inhibiting the uptake of advanced qMRI in clinics. With the proposed framework, parametric maps can be incorporated into clinical workflows and reporting systems, so that advanced qMRI could form a part of multimodal medical diagnoses and monitoring, without requiring cumbersome offline processing and data exports or imports. Although other existing methodologies could support this (e.g., Gadgetron [22, 23]), they are often still considered research tools, requiring additional computing infrastructures. With a repository of pretrained NNs, our strategy has no additional demand for local hardware resources or specialized expertise, offering the closest possible and least disruptive clinical integration of advanced qMRI. Further, immediate delivery of quantitative parametric maps enables new applications for such techniques in acute and point‐of‐care settings. While NNs are independent of generic acquisitions parameters (e.g., spatial resolution or TR, although SNR should be similar between training and in vivo data), they depend on the diffusion encoding scheme, or generally, model‐related protocol parameters. Therefore, a new diffusion MRI protocol requires training a new NN, though the ICE program is generalized to any diffusion MRI protocol and does not require recompilation. Providing a new NN would be akin to uploading new files of *b*‐vectors and *b*‐values onto the scanner PC. As NN training is relatively fast and utilizes synthetic data, it is not a burdensome procedure; indeed, scanner‐integrated training of NN_GT_ networks could be a future way of streamlining new protocol generation. However, the ICE program does not immediately generalize to any arbitrary qMRI modality; this would require source code modifications specific to the desired application.

Visual inspection of in vivo parametric maps shows similar outputs for NN and conventional MLE estimates, although minor quantitative differences, for example, increased NN FWF in white matter, are detectable. While overestimation of FWF is evident in scatter plots of Figure 4, it is less apparent in NN evaluations with synthetic data in Figure S4, especially for NN_MLE_, suggesting that either mismatches between training and in vivo SNR, or ‘out‐of‐model’ effects, like data quality issues, cause this discrepancy. The real‐world manifestation of noise is only partially addressed by training on Rician‐distributed measurements, since multi‐channel array coils and parallel imaging typically result in more complex noise behaviors, such as spatially varying noise levels [29]. While utilizing the adaptive combine algorithm for channel combination results in Rician‐distributed measurements [30], the ‘SENSE1’ combination strategy [31] (not available to us) may prove to be more optimal for our application. The discrepancy of FWF also improves slightly for the three‐shell protocol with additional diffusion encodings.

Otherwise, in vivo overestimations of ODI, or of NDI by NN_GT_, also manifest in scatter plots of synthetic data estimates in Figure S4, suggesting that they are of systematic origin. Further refinement of training data distributions, NN architecture and settings must take place to reduce these biases. As training the NN involves balancing accuracy across three interdependent parameters, and gains in one may lead to losses in another, one strategy might involve training separate networks to estimate each parameter. Crucially, in vivo MLE estimates serve only as benchmark or reference, not as ground truth. For discrepancies that do not manifest in synthetic data, it cannot be concluded whether MLE or NN estimates are more accurate.

Diffusion MRI has been known for its sensitivity to image artifacts and data quality issues, like geometric distortions and misalignments, caused by subject motion, magnetic susceptibility and eddy current effects [32]. Eddy currents are considered more problematic for diffusion modeling, as they directly arise from the application of strong diffusion gradients, and therefore produce variable misalignments across diffusion encodings [33]. In our case, this could lead to ‘out‐of‐model’ effects unseen to NNs. However, on modern scanner systems with state‐of‐the‐art eddy current compensation strategies and the highest possible EPI readout bandwidths, even *b* = 2000 s/mm^2^ images appear minimally corrupted by eddy currents. Shorter scan times achieved with acceleration techniques have reduced motion artifacts, too. Still, protocols for highly advanced diffusion models, such as soma and neurite density imaging (SANDI) [34], may require longer acquisitions and stronger diffusion encodings, at which stage residual eddy current effects and motion must be corrected for in post‐processing. With this proof‐of‐concept study, we demonstrate the capabilities of a minimally‐pre‐processed workflow which may be adequate for certain applications. To cover more use cases in the future, we plan to integrate as many pre‐processing steps as possible into a state‐of‐the‐art inline processing pipeline. A number of groups have already proposed susceptibility distortion [35, 36], head motion and eddy‐current distortion correction techniques [37] based on deep learning, which promise to be fast enough for inline implementation. It is already possible to perform inline denoising using vendor‐provided methods, such as Deep Resolve Gain.

From evaluations of synthetic test data in Figures S4 and S5, it is clear that NN_MLE_ produces estimates closer to the GT and to MLE estimates than NN_GT_. This is in agreement with Epstein et al., who proposed and demonstrated the MLE‐label framework for the intravoxel incoherent motion (IVIM) model [7]. We report that the strategy extends to more complex microstructure models, like NODDI, although estimation bias must be interpreted cautiously in the presence of parameter degeneracies. The framework offers a powerful and practical ML solution that emulates conventional fitting, with smaller additional biases. This is particularly crucial for atypical parameter distributions in disease.

## 5 Conclusion

In a proof‐of‐principle work, we demonstrate that real‐time, inline NODDI is possible with scanner‐integrated ML. Further, we have shown that a novel strategy to reduce parameter estimation bias and emulate conventional fitting with supervised ML extends well to a complex model like NODDI. These powerful strategies should generalize to any qMRI model and scanner vendor offering development capabilities on their reconstruction platform. This work represents a significant step in bringing qMRI techniques closer to clinical utility, delivering full integration of parametric maps with clinical workflows. The next steps will involve inline incorporation of common pre‐processing steps, and demonstration and validation in pathology. Outside of the clinic, real‐time data quality monitoring can be impactful even in research settings, accelerating and streamlining protocol development and optimization.

## Funding

This work was supported by National Institutes of Health, 1R01MH130362; University College London Hospitals Biomedical Research Centre; UK Research and Innovation, MR/S032290/1; Engineering and Physical Sciences Research Council, EP/S021930/1, EP/X525649/1.

## Conflicts of Interest

I.D. and C.T. are employees of Siemens Healthineers.

## Supporting information


**Table S1.** A summary of the diffusion MRI protocol parameters utilized for data synthesis and the in vivo imaging experiments. The first protocol utilizes the example two‐shell diffusion scheme of the NODDI MATLAB Toolbox. Diffusion vectors were distributed isotropically, *b* = 0 s/mm^2^ conditions were interleaved throughout and shells were sampled separately and consecutively in time. The second protocol is a three‐shell diffusion scheme with isotropically distributed diffusion vectors and interleaving of diffusion shells in time.
**Table S2.** A summary of neural network (NN) architecture and training settings.
**Table S3.** Brain‐regional means (μ) and standard deviations (σ) of in vivo estimated orientation dispersion index (ODI), neurite density index (NDI) and free water fraction (FWF) for all estimation methods (conventional MLE, NN_MLE_, NN_GT_), volunteers, rescans (RS) and protocols.
**Figure S1.** Estimated orientation dispersion index (ODI), neurite density index (NDI) and free water fraction (FWF) parameter maps for an axial slice of V2 and the three‐shell protocol. The upper row shows maps fitted conventionally with the NODDI MATLAB Toolbox (indicated MLE); the next two rows show maps inferred with the two trained neural networks (NN_MLE_ and NN_GT_). The bottom two rows show scatter plots of estimated parameters for each tissue type of single axial slices of all volunteers and rescans using the two‐shell protocol, comparing MLE to NN_MLE_ (row 4) and NN_GT_ (row 5). Pairwise MLE‐NN mean absolute differences for each tissue class are noted in the top left of each panel. NN parameter maps were exported as DICOMs, converted to NIFTI format and rescaled from integer (range 0–1000) to float (range 0–1). No post‐processing was performed.
**Figure S2.** Estimated orientation dispersion index (ODI), neurite density index (NDI) and free water fraction (FWF) parameter maps for an axial slice of all volunteers, rescans and protocols, computed with the neural network trained on conventionally estimated training labels (NN_MLE_).
**Figure S3.** Boxplots of estimated orientation dispersion index (ODI), neurite density index (NDI) and free water fraction (FWF) parameters for an axial slice across all volunteers and rescans (different colors) of the three‐shell protocol. Each panel shows data for a particular tissue type and estimation method, as indicated in row and column labels. The solid horizontal line indicates the median, the box indicates the interquartile range (IQR) and whiskers indicate outlier bounds, 1.5 IQRs from the lower and upper quartiles.
**Figure S4.** Results from offline model evaluation for the two‐shell protocol. The first two rows show 2D vector plots of the bias (relative to ground truths) in estimated parameters of synthetic test signals for the NN_MLE_ (red) and NN_GT_ (blue) networks, compared to a conventional maximum likelihood estimator (MLE) fit (black). Mean magnitude biases (3D) are displayed in central plots. The middle row shows standard deviations of estimated parameters. Marginalization was applied across parameter space for the visualizations of the bias (1D marginalization) and standard deviation (2D marginalization). The bottom two rows show scatter plots comparing NNs with MLE. To improve clarity, only estimates for the first 10 out of 100 repeats per parameter combination are shown. The mean of absolute differences (|Δ|) calculated across all 100 repeats is indicated.
**Figure S5.** Brain Results from offline model evaluation for the three‐shell protocol. The first two rows show 2D vector plots of the bias (relative to ground truths) in estimated parameters of synthetic test signals for the NN_MLE_ (red) and NN_GT_ (blue) networks, compared to a conventional maximum likelihood estimator (MLE) fit (black). Mean magnitude biases (3D) are displayed in central plots. The middle row shows standard deviations of estimated parameters. Marginalization was applied across parameter space for the visualizations of the bias (1D marginalization) and standard deviation (2D marginalization). The bottom two rows show scatter plots comparing NNs with MLE. To improve clarity, only estimates for the first 10 out of 100 repeats per parameter combination are shown. The mean of absolute differences (|Δ|) calculated across all 100 repeats is indicated.

## Data Availability

Code to support and reproduce this work relies on an internal version of the NODDI MATLAB Toolbox (NMT) and will be made publicly available as part of the next NMT release. Additional methodological details are available from the authors upon reasonable request.
