# Supplementary material for: Real‐Time, Inline Quantitative MRI Enabled by Scanner‐Integrated Machine Learning: A Proof of Principle With NODDI
Source: Magn Reson Med. 2026 May 5;96(2):986–95. doi: 10.1002/mrm.70388 (PMC13269190; doi:10.1002/mrm.70388)
Supplement: Supplementary file 1 — Table S1. A summary of the diffusion MRI protocol parameters utilized for data synthesis and the in vivo imaging experiments. The first protocol utilizes the example two‐shell diffusion scheme of the NODDI MATLAB Toolbox. Diffusion vectors were distributed isotropically, b = 0 s/mm2 conditions were interleaved throughout and shells were sampled separately and consecutively in time. The second protocol is a three‐shell diffusion scheme with isotropically distributed diffusion vectors and interleaving of diffusion shells in time. Table S2. A summary of neural network (NN) architecture and training settings. Table S3. Brain‐regional means (μ) and standard deviations (σ) of in vivo estimated orientation dispersion index (ODI), neurite density index (NDI) and free water fraction (FWF) for all estimation methods (conventional MLE, NNMLE, NNGT), volunteers, rescans (RS) and protocols. Figure S1. Estimated orientation dispersion index (ODI), neurite density index (NDI) and free water fraction (FWF) parameter maps for an axial slice of V2 and the three‐shell protocol. The upper row shows maps fitted conventionally with the NODDI MATLAB Toolbox (indicated MLE); the next two rows show maps inferred with the two trained neural networks (NNMLE and NNGT). The bottom two rows show scatter plots of estimated parameters for each tissue type of single axial slices of all volunteers and rescans using the two‐shell protocol, comparing MLE to NNMLE (row 4) and NNGT (row 5). Pairwise MLE‐NN mean absolute differences for each tissue class are noted in the top left of each panel. NN parameter maps were exported as DICOMs, converted to NIFTI format and rescaled from integer (range 0–1000) to float (range 0–1). No post‐processing was performed. Figure S2. Estimated orientation dispersion index (ODI), neurite density index (NDI) and free water fraction (FWF) parameter maps for an axial slice of all volunteers, rescans and protocols, computed with the neural network trained on conventionally e [file MRM-96-986-s001.docx]

**SUPPORTING INFORMATION**

**Real-time, inline quantitative MRI enabled by scanner-integrated machine learning: a proof of principle with NODDI**

**Table S1**

A summary of the diffusion MRI protocol parameters utilised for data synthesis and the in vivo imaging experiments. The first protocol utilises the example two-shell diffusion scheme of the NODDI MATLAB Toolbox. Diffusion vectors were distributed isotropically, $b=0$ s/mm^2^ conditions were interleaved throughout and shells were sampled separately and consecutively in time. The second protocol is a three-shell diffusion scheme with isotropically distributed diffusion vectors and interleaving of diffusion shells in time.

| Multi-shell diffusion imaging protocols | |
| --- | --- |
| Sequence | EPI spin echo |
| *b* values (nr. encodings) (s/mm^2^) |  |
| Two-shell protocol | 0 (9), 700 (24), 2000 (48) |
| Three-shell protocol | 0 (12), 300 (8), 700 (32), 2000 (64) |
| TE (ms) | 83 |
| TR (ms) | 2900 |
| FOV (mm^2^) | 200x200 |
| Voxel size (mm^2^) | 2x2 |
| Nr. slices |  |
| Two-shell protocol | 40 |
| Three-shell protocol | 50 |
| Slice thickness (mm) | 2 |
| Bandwidth (Hz/px) | 2000 |
| GRAPPA factor, reference lines | 2, 20 |
| Simultaneous multi-slice (SMS) factor | 2 |
| Partial Fourier | 7/8 |
| Gradient scheme | Monopolar |
| Scan time (mm:ss) |  |
| Two-shell protocol | 04:09 |
| Three-shell protocol | 05:52 |
| Channel combination | Adaptive combine |

**Table S2**

A summary of neural network (NN) architecture and training settings.

| NN architecture | |
| --- | --- |
| Network type | Fully connected feed-forward NN |
| Input nodes | 81 or 116 |
| Hidden layers | 3 |
| Hidden nodes | 120 (per layer) |
| Hidden activation | ELU (α = 0.05) |
| Output nodes | 3 |
| Output activation | Hard sigmoid |
| Trainable parameters | 39243 or 43443 |
| NN training | |
| Python library | PyTorch (version 2.1.1) |
| Batch size | 1000 |
| Epochs | 500 |
| Validation fraction | 0.1 |
| Loss function | Mean squared error |
| Optimiser | Adam |
| Learning rate | 5e-4 |
| L2 regularisation strength | 1e-6 |
| Momentum | 0.9 |
| GPU chip | NVIDIA GeForce RTX 4090 (CUDA 12.1) |
| Training time (mm:ss) | ~ 10:00 |

**Table S3**

Brain-regional means ($\mu$) and standard deviations ($\sigma$) of in vivo estimated orientation dispersion index (ODI), neurite density index (NDI) and free water fraction (FWF) for all estimation methods (conventional MLE, NN_MLE_, NN_GT_), volunteers, rescans (RS) and protocols.

| **Subject** | **Protocol** | **Method** | **Tissue** | **Nr. voxels** | **ODI** | **NDI** | **FWF** |
| --- | --- | --- | --- | --- | --- | --- | --- |
|  | **(nr. shells)** |  |  |  | $\mu\pm\sigma$ | $\mu\pm\sigma$ | $\mu\pm\sigma$ |
| V1 | 2 | MLE | WM | 1022 | 0.18 ± 0.10 | 0.59 ± 0.11 | 0.10 ± 0.09 |
| V2 | 2 | MLE | WM | 1289 | 0.18 ± 0.10 | 0.60 ± 0.11 | 0.09 ± 0.08 |
| V2 RS | 2 | MLE | WM | 1285 | 0.18 ± 0.10 | 0.62 ± 0.11 | 0.09 ± 0.08 |
| V1 | 3 | MLE | WM | 1022 | 0.18 ± 0.10 | 0.58 ± 0.10 | 0.08 ± 0.07 |
| V2 | 3 | MLE | WM | 1302 | 0.18 ± 0.10 | 0.62 ± 0.10 | 0.09 ± 0.07 |
| V2 RS | 3 | MLE | WM | 1337 | 0.18 ± 0.10 | 0.61 ± 0.09 | 0.09 ± 0.07 |
| V1 | 2 | NN_MLE_ | WM | 1022 | 0.21 ± 0.09 | 0.61 ± 0.11 | 0.18 ± 0.09 |
| V2 | 2 | NN_MLE_ | WM | 1289 | 0.21 ± 0.09 | 0.62 ± 0.10 | 0.17 ± 0.07 |
| V2 RS | 2 | NN_MLE_ | WM | 1285 | 0.21 ± 0.09 | 0.64 ± 0.10 | 0.18 ± 0.07 |
| V1 | 3 | NN_MLE_ | WM | 1022 | 0.21 ± 0.09 | 0.59 ± 0.09 | 0.14 ± 0.07 |
| V2 | 3 | NN_MLE_ | WM | 1302 | 0.20 ± 0.09 | 0.64 ± 0.10 | 0.15 ± 0.07 |
| V2 RS | 3 | NN_MLE_ | WM | 1337 | 0.20 ± 0.09 | 0.63 ± 0.09 | 0.15 ± 0.06 |
| V1 | 2 | NN_GT_ | WM | 1022 | 0.22 ± 0.10 | 0.63 ± 0.11 | 0.18 ± 0.07 |
| V2 | 2 | NN_GT_ | WM | 1289 | 0.21 ± 0.10 | 0.63 ± 0.10 | 0.17 ± 0.06 |
| V2 RS | 2 | NN_GT_ | WM | 1285 | 0.21 ± 0.10 | 0.65 ± 0.10 | 0.17 ± 0.05 |
| V1 | 3 | NN_GT_ | WM | 1022 | 0.20 ± 0.10 | 0.59 ± 0.09 | 0.14 ± 0.05 |
| V2 | 3 | NN_GT_ | WM | 1302 | 0.19 ± 0.09 | 0.62 ± 0.10 | 0.15 ± 0.05 |
| V2 RS | 3 | NN_GT_ | WM | 1337 | 0.20 ± 0.10 | 0.62 ± 0.09 | 0.15 ± 0.05 |
| V1 | 2 | MLE | GM | 251 | 0.44 ± 0.14 | 0.42 ± 0.12 | 0.03 ± 0.11 |
| V2 | 2 | MLE | GM | 272 | 0.45 ± 0.11 | 0.46 ± 0.09 | 0.01 ± 0.03 |
| V2 RS | 2 | MLE | GM | 275 | 0.45 ± 0.11 | 0.46 ± 0.10 | 0.01 ± 0.06 |
| V1 | 3 | MLE | GM | 215 | 0.47 ± 0.13 | 0.44 ± 0.11 | 0.01 ± 0.08 |
| V2 | 3 | MLE | GM | 258 | 0.45 ± 0.11 | 0.47 ± 0.11 | 0.01 ± 0.08 |
| V2 RS | 3 | MLE | GM | 251 | 0.46 ± 0.11 | 0.46 ± 0.10 | 0.01 ± 0.07 |
| V1 | 2 | NN_MLE_ | GM | 251 | 0.45 ± 0.14 | 0.39 ± 0.12 | 0.06 ± 0.11 |
| V2 | 2 | NN_MLE_ | GM | 272 | 0.48 ± 0.11 | 0.40 ± 0.05 | 0.02 ± 0.06 |
| V2 RS | 2 | NN_MLE_ | GM | 275 | 0.48 ± 0.12 | 0.41 ± 0.05 | 0.03 ± 0.07 |
| V1 | 3 | NN_MLE_ | GM | 215 | 0.50 ± 0.13 | 0.38 ± 0.09 | 0.03 ± 0.08 |
| V2 | 3 | NN_MLE_ | GM | 258 | 0.48 ± 0.12 | 0.39 ± 0.07 | 0.02 ± 0.08 |
| V2 RS | 3 | NN_MLE_ | GM | 251 | 0.48 ± 0.12 | 0.38 ± 0.05 | 0.02 ± 0.07 |
| V1 | 2 | NN_GT_ | GM | 251 | 0.54 ± 0.14 | 0.44 ± 0.09 | 0.10 ± 0.11 |
| V2 | 2 | NN_GT_ | GM | 272 | 0.55 ± 0.14 | 0.42 ± 0.05 | 0.06 ± 0.05 |
| V2 RS | 2 | NN_GT_ | GM | 275 | 0.55 ± 0.15 | 0.42 ± 0.05 | 0.06 ± 0.07 |
| V1 | 3 | NN_GT_ | GM | 215 | 0.57 ± 0.15 | 0.41 ± 0.07 | 0.06 ± 0.09 |
| V2 | 3 | NN_GT_ | GM | 258 | 0.53 ± 0.15 | 0.40 ± 0.05 | 0.04 ± 0.08 |
| V2 RS | 3 | NN_GT_ | GM | 251 | 0.55 ± 0.15 | 0.40 ± 0.04 | 0.05 ± 0.07 |
| V1 | 2 | MLE | CSF | 145 |  |  | 0.97 ± 0.11 |
| V2 | 2 | MLE | CSF | 91 |  |  | 0.97 ± 0.13 |
| V2 RS | 2 | MLE | CSF | 95 |  |  | 0.97 ± 0.14 |
| V1 | 3 | MLE | CSF | 145 |  |  | 0.97 ± 0.10 |
| V2 | 3 | MLE | CSF | 93 |  |  | 0.97 ± 0.15 |
| V2 RS | 3 | MLE | CSF | 87 |  |  | 0.98 ± 0.11 |
| V1 | 2 | NN_MLE_ | CSF | 145 |  |  | 0.95 ± 0.12 |
| V2 | 2 | NN_MLE_ | CSF | 91 |  |  | 0.96 ± 0.12 |
| V2 RS | 2 | NN_MLE_ | CSF | 95 |  |  | 0.95 ± 0.13 |
| V1 | 3 | NN_MLE_ | CSF | 145 |  |  | 0.92 ± 0.11 |
| V2 | 3 | NN_MLE_ | CSF | 93 |  |  | 0.93 ± 0.16 |
| V2 RS | 3 | NN_MLE_ | CSF | 87 |  |  | 0.91 ± 0.16 |
| V1 | 2 | NN_GT_ | CSF | 145 |  |  | 0.88 ± 0.08 |
| V2 | 2 | NN_GT_ | CSF | 91 |  |  | 0.89 ± 0.11 |
| V2 RS | 2 | NN_GT_ | CSF | 95 |  |  | 0.89 ± 0.10 |
| V1 | 3 | NN_GT_ | CSF | 145 |  |  | 0.85 ± 0.08 |
| V2 | 3 | NN_GT_ | CSF | 93 |  |  | 0.85 ± 0.14 |
| V2 RS | 3 | NN_GT_ | CSF | 87 |  |  | 0.88 ± 0.09 |

**Figure S1**

***Figure S1*** *Estimated orientation dispersion index (ODI), neurite density index (NDI) and free water fraction (FWF) parameter maps for an axial slice of V2 and the three-shell protocol. The upper row shows maps fitted conventionally with the NODDI MATLAB Toolbox (indicated MLE); the next two rows show maps inferred with the two trained neural networks (NN_MLE_ and NN_GT_). The bottom two rows show scatter plots of estimated parameters for each tissue type of single axial slices of all volunteers and rescans using the two-shell protocol, comparing MLE to NN_MLE_ (row 4) and NN_GT_ (row 5). Pairwise MLE-NN mean absolute differences for each tissue class are noted in the top left of each panel. NN parameter maps were exported as DICOMs, converted to NIFTI format and rescaled from integer (range 0 to 1000) to float (range 0 to 1). No post-processing was performed.*

**Figure S2**

***Figure S2*** *Estimated orientation dispersion index (ODI), neurite density index (NDI) and free water fraction (FWF) parameter maps for an axial slice of all volunteers, rescans and protocols, computed with the neural network trained on conventionally estimated training labels (NN_MLE_).*

**Figure S3**

***Figure S3*** *Boxplots of estimated orientation dispersion index (ODI), neurite density index (NDI) and free water fraction (FWF) parameters for an axial slice across all volunteers and rescans (different colours) of the three-shell protocol. Each panel shows data for a particular tissue type and estimation method, as indicated in row and column labels. The solid horizontal line indicates the median, the box indicates the interquartile range (IQR) and whiskers indicate outlier bounds, 1.5 IQRs from the lower and upper quartiles.*

**Figure S4**

***Figure S4*** *Results from offline model evaluation for the two-shell protocol. The first two rows show 2D vector plots of the bias (relative to ground truths) in estimated parameters of synthetic test signals for the NN_MLE_ (red) and NN_GT_ (blue) networks, compared to a conventional maximum likelihood estimator (MLE) fit (black). Mean magnitude biases (3D) are displayed in central plots. The middle row shows standard deviations of estimated parameters. Marginalisation was applied across parameter space for the visualisations of the bias (1D marginalisation) and standard deviation (2D marginalisation). The bottom two rows show scatter plots comparing NNs with MLE. To improve clarity, only estimates for the first 10 out of 100 repeats per parameter combination are shown. The mean of absolute differences (*$\bar{\left| \Delta\right|}$*) calculated across all 100 repeats is indicated.*

**Figure S5**

**

***Figure S5*** *Results from offline model evaluation for the three-shell protocol. The first two rows show 2D vector plots of the bias (relative to ground truths) in estimated parameters of synthetic test signals for the NN_MLE_ (red) and NN_GT_ (blue) networks, compared to a conventional maximum likelihood estimator (MLE) fit (black). Mean magnitude biases (3D) are displayed in central plots. The middle row shows standard deviations of estimated parameters. Marginalisation was applied across parameter space for the visualisations of the bias (1D marginalisation) and standard deviation (2D marginalisation). The bottom two rows show scatter plots comparing NNs with MLE. To improve clarity, only estimates for the first 10 out of 100 repeats per parameter combination are shown. The mean of absolute differences (*$\bar{\left| \Delta\right|}$*) calculated across all 100 repeats is indicated.*
